# Evaluation of Staff Experience of Dialogically Informed Training in a Community Mental Health Rehabilitation Team

**DOI:** 10.1192/bjo.2026.11334

**Published:** 2026-06-30

**Authors:** Jordan Burrell, Val Jackson, Connor Johnston, Amrith Shetty

**Affiliations:** 1 Cheshire & Wirral Partnership NHS Foundation Trust, Chester, United Kingdom; 2 Developing Open Dialogue, West Yorkshire, United Kingdom

## Abstract

**Aims:**

The Mental Health Intensive Support Team at Cheshire and Wirral Partnership NHS Foundation Trust commissioned a bespoke, 8-day training programme centred on the principles and delivery of dialogical practice, informed by an Open Dialogue approach. The training was commissioned to support the embedding of these principles into the rehabilitative model of care, with consideration being given to how to adapt the approach to the clinical model.

The aim was to evaluate the experiences of the clinical staff participating in the training that was delivered between April 2025 and November 2025, across two training cohorts.

**Methods:**

The training package was offered to clinical staff of all bandings who had not previously completed any formal training in Open Dialogue or dialogical practice.

Data was collected via questionnaires that were completed at key points throughout the training. A pre-training questionnaire was completed prior to starting the 8-day training programme, and a post-training questionnaire after each of the four 2-day training blocks. All questionnaires were optional and anonymous.

Data collected was collated and a reflexive thematic analysis was completed to identify core themes highlighting staff experiences.

**Results:**

Across both cohorts the self-rated knowledge scores consistently improved across each training block. Average scores went from 4/4.5 out of 10 prior to attending the training (cohort 1/cohort 2 respectively) to 8.6/8.4 out of 10 at the completion of training (cohort 1/cohort 2 respectively) across both cohorts. 100% of respondents felt that the training met their expectations, alongside 98% of respondents feeling that the training will improve their work.

A reflexive thematic analysis identified six core themes from the qualitative data: (1) feeling more comfortable – beginner to more competent, (2) the value of experiential learning, (3) growing through it, (4) learning together, (5) learning how to really listen and (6) empathetic hope for the future.

**Conclusion:**

The data identified that participants, overall, had a resoundingly positive experience of the training. Participants’ knowledge and understanding of dialogical principles improved, alongside participants also reporting positive changes both personally and relationally. The latter wasn’t an intended outcome but aligns with experiences of Open Dialogue training reported in the literature. The evaluation demonstrated that many participants left the training feeling hopeful for a future where dialogical principles areembedded into care delivery, and where individuals and their networks have more opportunities to have their voices heard.

